# Development and validation of a molecular prognostic index of bladder cancer based on immunogenomic landscape analysis

**DOI:** 10.1186/s12935-020-01343-3

**Published:** 2020-07-11

**Authors:** Ning Xu, Zhi-Bin Ke, Xiao-Dan Lin, Ye-Hui Chen, Yu-Peng Wu, Yu Chen, Ru-Nan Dong, Shao-Hao Chen, Xiao-Dong Li, Yong Wei, Qing-Shui Zheng, Yun-Zhi Lin, Xue-Yi Xue

**Affiliations:** 1 Department of Urology, The First Affiliated Hospital of Fujian Medical University, 20 Chazhong Road, Fuzhou, 350005 China; 2 Cancer Bio-immunotherapy Center, Fujian Medical University Cancer Hospital and Fujian Cancer Hospital, Fuzhou, China; 3 Department of Medical Oncology, Fujian Medical University Cancer Hospital and Fujian Cancer Hospital, Fuzhou, China

**Keywords:** Bladder cancer, Immune-related genes, Prognostic index, Survival outcome

## Abstract

**Background:**

Bladder cancer (BCa) is one of the important tumors that have been proven to be treatable with immunotherapy. This study aims to identify and validate a molecular prognostic index of BCa based on immunogenomic landscape analysis.

**Methods:**

The cancer genome atlas (TCGA) database and immunology database and analysis portal (ImmPort) database were used to identified differentially expressed immune-related genes (IRGs). Prognostic IRGs were screened and protein–protein interaction (PPI) network was constructed. Multivariate Cox analysis was performed to develop a molecular prognostic index of BCa. Internal and external validation were then performed in TCGA cohort and GEO cohort, respectively. Besides, we also explore the relationship between this index and clinical characteristics, immune cell infiltration and tumor microenvironment.

**Results:**

A total of 61 prognostic IRGs were identified and a molecular prognostic index was developed. The top four hub genes included MMP9, IGF1, CXCL12 and PGF. The difference in overall survival between high-risk group and low-risk group was statistically significant. The area under curve of the receiver operating characteristic (ROC) curve was 0.757, suggesting the potential for this index. Besides, Internal validation using TCGA cohort and external validation using GEO cohort indicated that this index was of great performance in predicting outcome. T cells CD8, T cells CD4 memory activated, T cells follicular helper, macrophages M0, macrophages M2 and neutrophils were significantly associated with prognosis of BCa patients. Female, high grade, stage III&IV, N1-3 and T3-4 were associated significantly with higher risk score compared with male, low grade, stage I&II, N0 and T1-2, respectively. High risk score had a positive association with higher stromal score and ESTIMATE score while high risk score had a negative association with tumor purity.

**Conclusions:**

This study identified several prognostic immune-related genes of clinical value. Besides, we developed and validated a molecular index based on immunogenomic landscape analysis, which performed well in predicting prognosis of BCa. Further researches are needed to verify the effectiveness of this index and these vital genes.

## Background

Bladder cancer (BCa) was very common and regarded as the sixth most frequent cause of mortality related to malignancy [1, 2]. Radical cystectomy remains the standard treatment of muscle invasive bladder cancer [3]. However, most patients require postoperative adjuvant therapy according to latest guideline [4]. The application of adjuvant chemotherapy significantly improved prognosis in patients with BCa [4, 5]. Nowadays, immunotherapy is considered as a nonnegligible treatment for solid malignancies by strengthening the immune system against tumors [6, 7]. BCa is one of the important tumors that have been proven to be treatable with immunotherapy [6]. Since 1976, intravesical instillation of Bacillus Calmette-Guérin (BCG) has been widely used in the treatment of BCa [8]. Recently, with the introduction of checkpoint inhibitors into clinical practice, immunotherapy plays a more important role in anti-tumor therapy in BCa patients, particularly those who were refractory to conventional treatment [9]. Therefore, it is of great importance to explore the immune components and relevant mechanisms.

This study aimed to identified the immune-related genes (IRGs), especially prognostic IRGs, in BCa microenvironment using bioinformatics methods. We also explored the underlying clinical application of IRGs on prognostic stratification. Importantly, we constructed a molecular prognostic index based on these IRGs and explored the relationship between the prognostic index and immune cell infiltration, clinical characteristics and tumor microenvironment.

## Methods

### Data acquisition

We downloaded transcriptome data and clinical data of 412 BCa samples and 19 normal samples from the Cancer Genome Atlas (TCGA) database (https://tcga-data.nci.nih.gov/tcga/). Besides, external validation data were extracted from GSE19423 and GSE32894 dataset in Gene Expression Omnibus (GEO) database (https://www.ncbi.nlm.nih.gov/geo/). The immune-related genes (IRGs), which have been confirmed to play a vital role in immune activity, were identified from Immunology Database and Analysis Portal (ImmPort) database (https://www.immport.org/).

### Identification of differentially expressed IRGs

The transcriptome data from TCGA database and GEO database was analyzed using R x64 3.6.1 software (https://www.r-project.org/). The R package limma and Wilcox test were applied to filtrate the differentially expressed IRGs for further analysis. The cut-off value was false discovery rate (FDR) < 0.01 and log2|fold change (FC)| > 1. Importantly, univariate Cox regression analysis was used to extract prognosis-associated differentially expressed IRGs of BCa, and P < 0.05 was considered statistically significant.

### Functional analysis of differentially expressed IRGs

Gene ontology analysis (GO) is applied to annotate differentially expressed IRGs. The results of GO analysis were presented by three parts including biological processes (BP), molecular functions (MF), and cellular component (CC). Besides, the Kyoto Encyclopedia of Genes and Genomes (KEGG) analysis was used to perform the pathway enrichment analysis. Both GO analysis and KEGG analysis were conducted using R x64 3.6.1 software.

### Construction of protein–protein interaction (PPI) network and hub genes selection

In this step, we constructed PPI network of prognosis-associated differentially expressed IRGs that have been identified in previous analysis. Search Tool for the Retrieval of Interacting Genes (STRING) database (version 11.0; https://string-db.org/cgi/input.pl) was used to evaluate the PPI information. Cytoscape software (version 3.6.1) was used to visualize the PPI networks and select hub genes for further discussion.

### Development of the IRGs-based prognostic index

By using multivariate Cox regression analysis, we established a prognostic index based on these differentially expressed IRGs, which has significant association with the survival of BCa patients. Finally, patients were divided into two groups, high-risk group and low-risk group, according to median value of the risk score. Survival analysis and the receiver operating characteristic (ROC) curve was performed to validate the performance of the index. Besides, independent prognostic analysis was used to evaluated whether this index is an independent prognostic factor of overall survival (OS).

### Internal and external validation of the IRGs-based prognostic index

We validated this prognostic index in external independent database. We also selected survival-related IRGs from GEO database. The index was then validated externally in GEO cohort. The patients were also divided in high risk group and low risk group. And the survival analysis was performed to conform the usability of this prognostic index. Furthermore, in order to perform internal validation of this index, the patients from TCGA cohort were randomly divided into train group and test group. We then performed survival analysis in train group and test group, respectively.

### Evaluation of relationship between this prognostic index and immune cell infiltration, clinical characteristics and tumor microenvironment

The Tumor Immune Estimation Resource (TIMER) database version 2.0 (https://cistrome.shinyapps.io/timer/) was used to estimate the relationship between this index and 22 subtypes of tumor-infiltrating immune cells. Besides, we also explore the relationship between this index and clinical characteristics obtained from TCGA databases including age, gender, grader, stage, T, N and M stage. Tumor microenvironment has been regarded as an important factor which plays a vital role in carcinogenesis. ESTIMATE was an algorithm for estimating immune score, stromal score and tumor purity in tumor microenvironment [10]. In this study, we calculate immune score, stromal score and tumor purity score using ESTIMATE algorithm to explore the relationship between this index and tumor microenvironment. P value < 0.05 was considered statistically significant. Statistical analyses were performed using R software.

## Results

### Identification of differentially expressed IRGs

**Fig. 1:**
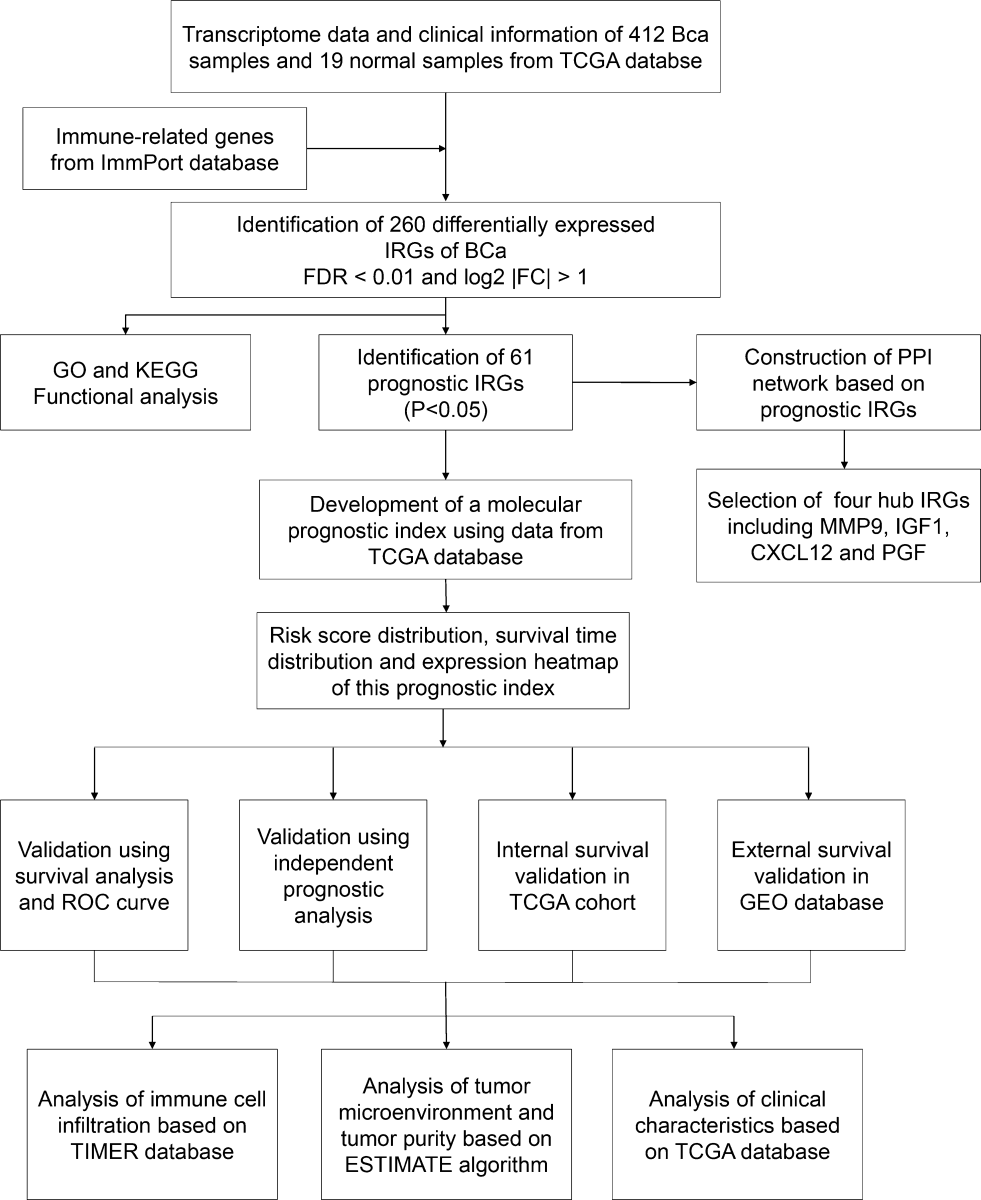
The flow diagram of this study

A total of 412 BCa samples and 19 normal samples from TCGA were included in this study and 4876 differentially expressed genes (DEGs) between BCa tissue and normal tissue were identified. The clinicopathological characteristic of 412 patients with BCa were showed in Table 1. Then, 2498 IRGs were extracted from ImmPort database, among which 260 differentially expressed IRGs were filtrated for further analysis. The flow diagram of this study was showed in Fig. 1.

**Table 1:** Clinicopathological characteristic of 412 patients with BCa from TCGA database

Clinicopathological characteristics	Value
Age, years	
Mean ± SD	68.09 ± 10.58
Range	34–90
Gender, n (%)	
Female	108 (26.2)
Male	304 (73.8)
Grade, n (%)	
High	388 (94.1)
Low	21 (5.1)
Unknown	3 (0.8)
TCGA stage	
Stage I	2 (0.5)
Stage II	131 (31.8)
Stage III	141 (34.2)
Stage IV	136 (33.0)
Unknown	2 (0.5)
T stage, n (%)	
T0	1 (0.2)
T1	3 (0.7)
T2	120 (29.1)
T3	196 (47.6)
T4	59 (14.3)
Unknown	33 (8.1)
N stage, n (%)	
N0	239 (58.1)
N1	47 (11.4)
N2	76 (18.4)
N3	8 (1.9)
Unknown	42 (10.2)
M stage, n (%)	
M0	196 (47.5)
M1	11 (2.7)
Unknown	205 (49.8)

### Functional analysis of differentially expressed IRGs

**Fig. 2:**
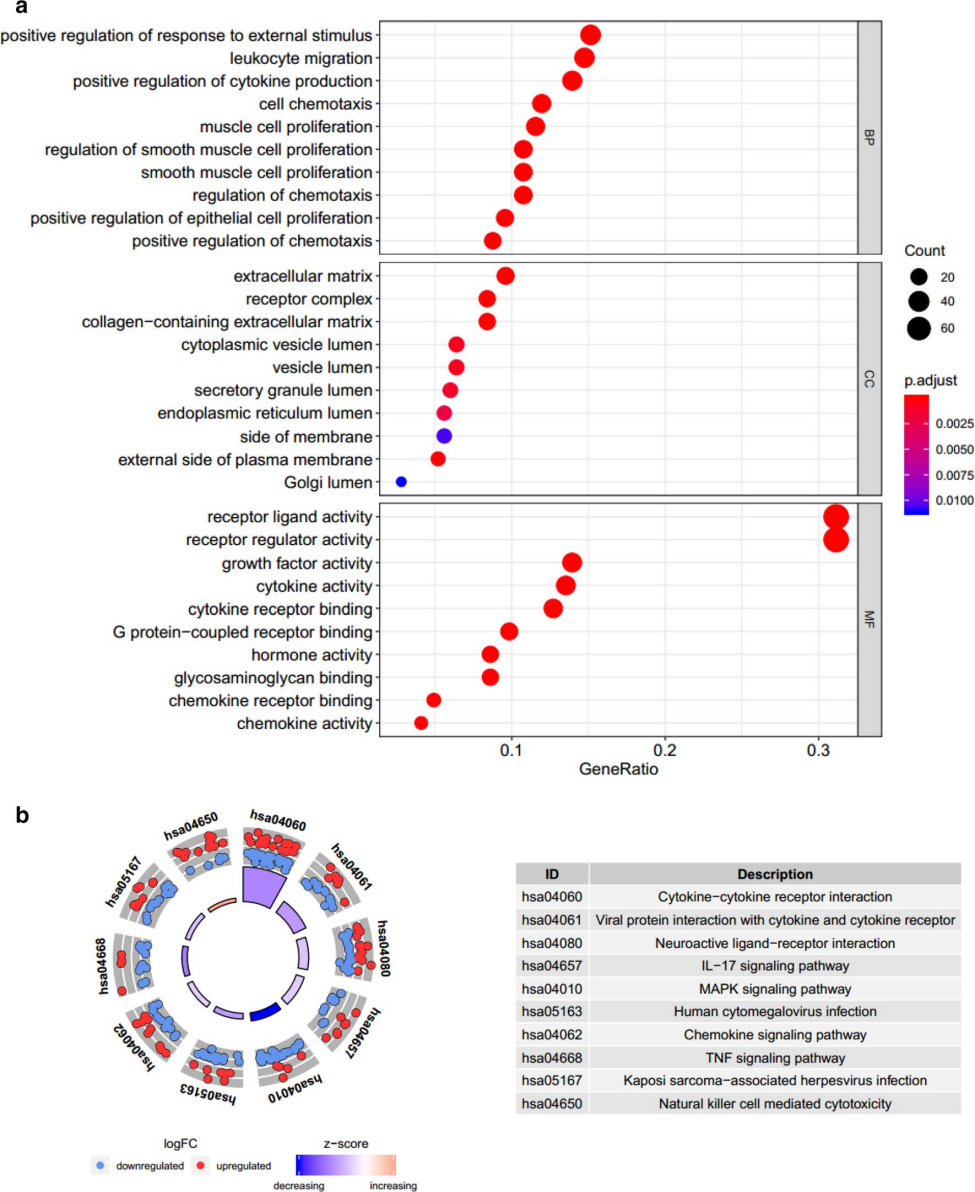
Genes ontology (GO) analysis of 260 differentially expressed IRGs (**a**). Enriched pathways of 260 differentially expressed IRGs according to Kyoto Encyclopedia of Genes and Genomes (KEGG) analysis (**b**)

GO analysis related to BP revealed that these differentially expressed IRGs were mainly involved in positive regulation of response to external stimulus. GO analysis related to CC showed that these differentially expressed IRGs were mainly enriched in extracellular matrix and receptor complex. GO analysis related to MF demonstrated that these differentially expressed IRGs were involved in receptor ligand activity (Fig. 2a). KEGG analysis showed that cytokine–cytokine receptor interaction was the most important pathway (Fig. 2b).

### Identification of prognostic IRGs

**Fig. 3:**
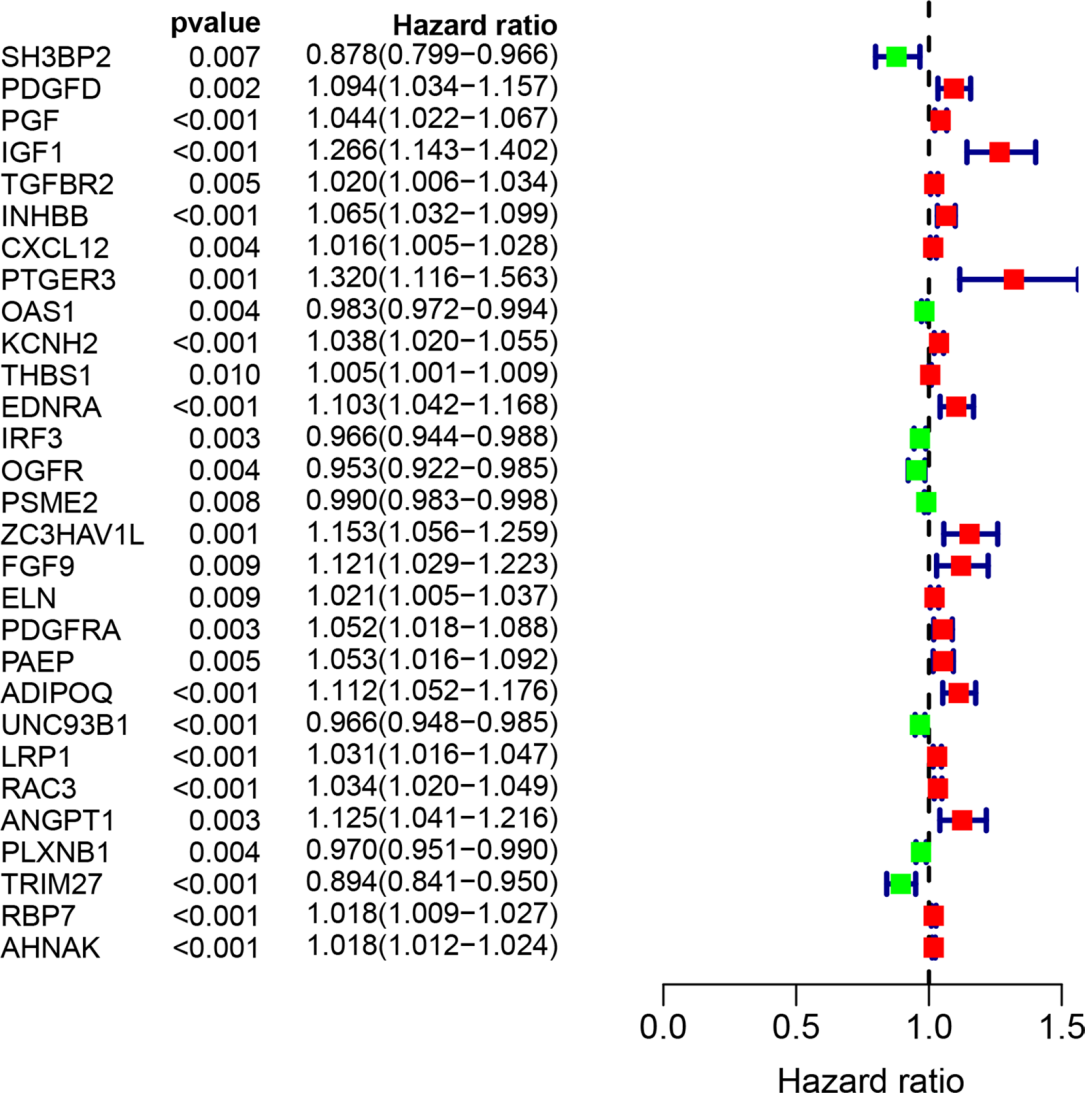
Univariate Cox regression analysis identifying prognostic differentially expressed IRGs

A total of 61 prognostic differentially expressed IRGs were identified using univariate Cox model (P < 0.05). There is a significant correlation between these 61 genes and OS; therefore, they were extracted for further study. The most significant genes were presented in Fig. 3. According to the forest plot of hazard ratios, most of these genes were risk factors for poor prognosis in bladder cancer patients. That is to say, the higher the expression of these genes which were presented by red node, the higher the probability of poor prognosis.

### Construction of PPI network based on prognostic IRGs

**Fig. 4:**
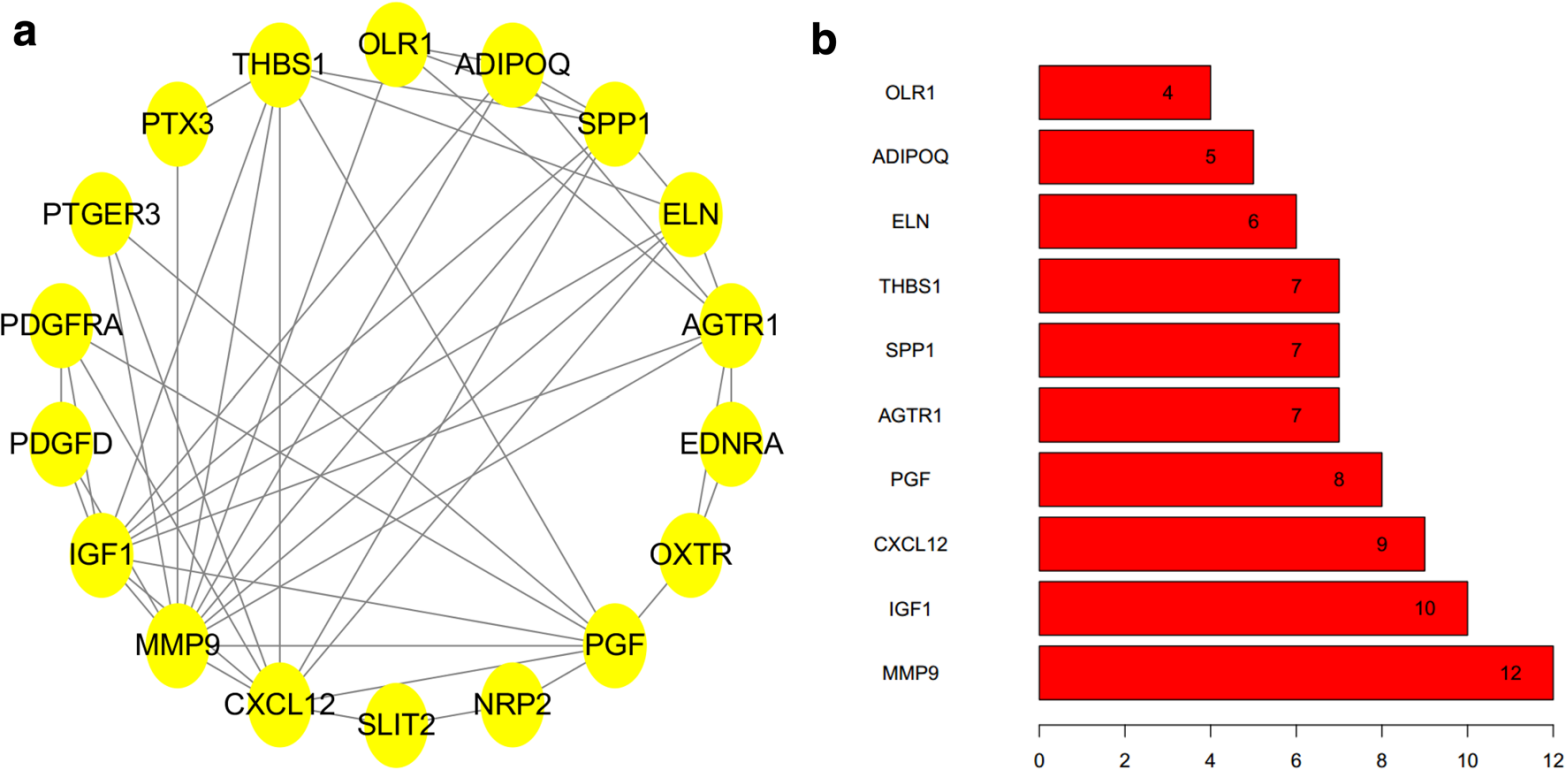
Construction of protein–protein interaction (PPI) network based on prognostic IRGs (**a**). the top 10 genes with highest degree scores in PPI network (**b**)

Proteins related to prognostic differentially expressed IRGs were selected based on STRING and visualized by Cytoscape version 3.6.1 (Fig. 4a). Furthermore, in this network, the top four genes with highest degree scores were selected as hub IRGs including matrix metallopeptidase 9 (MMP9), insulin like growth factor 1 (IGF1), C-X-C motif chemokine ligand 12 (CXCL12) and placental growth factor (PGF) (Fig. 4b).

### Development of a molecular prognostic index

**Fig. 5:**
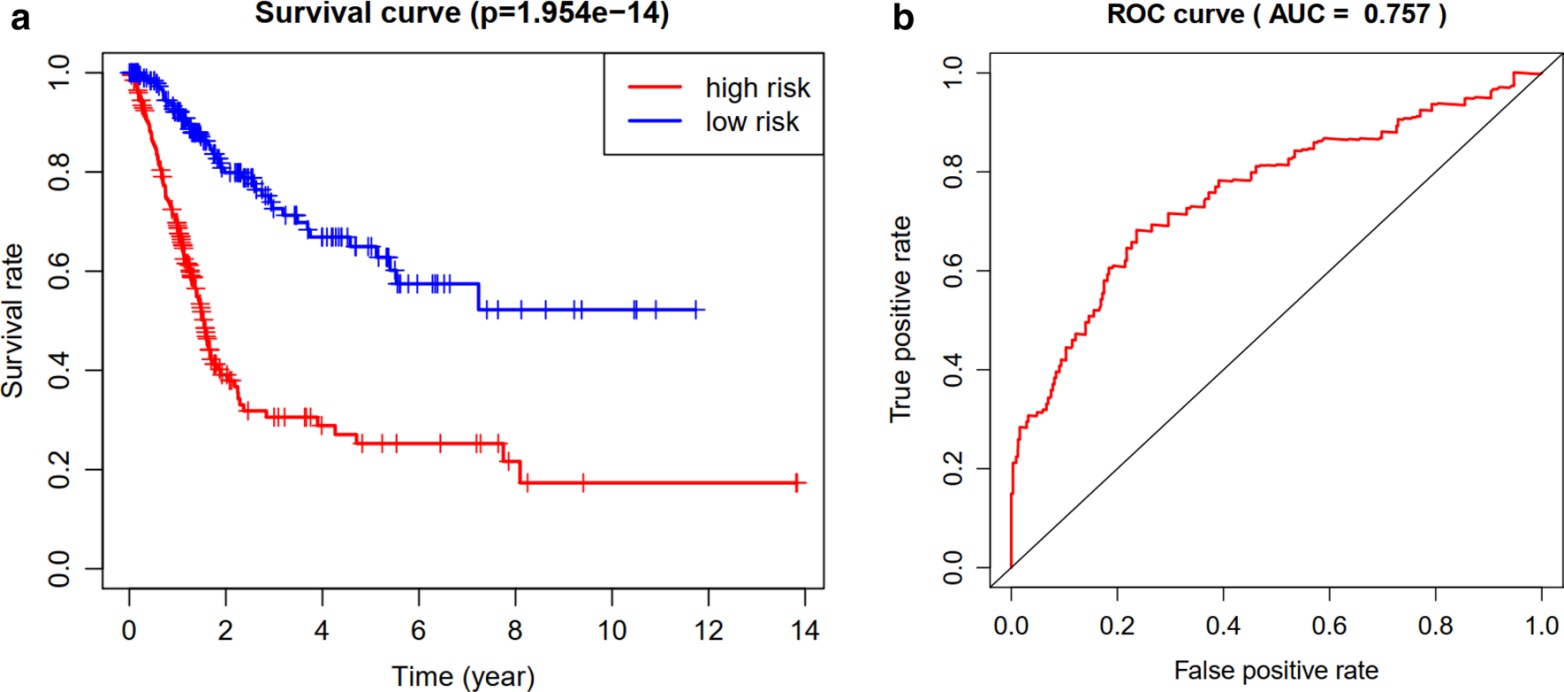
Comparison of overall survival (OS) between high-risk group and low-risk group in TCGA cohort (**a**). The receiver operating characteristic (ROC) curve of this index in TCGA cohort (**b**)

Then, we developed a molecular prognostic index based on these differentially expressed prognostic IRGs (Table 2). The difference in overall survival between high-risk group and low-risk group was statistically significant (P < 0.05) (Fig. 5a). The area under curve of ROC was 0.757, suggesting the potential for the prognostic index (Fig. 5b).

**Table 2:** Multivariate cox analysis to developing a prognostic index based on these differentially expressed IRGs

Id	Coef	HR	HR.95L	HR.95H	P value
FCN2	− 0.38509671	0.680384831	0.410057411	1.128923671	0.136069967
ISG15	− 0.00172587	0.998275622	0.996968943	0.999584013	0.00980666
ANXA6	− 0.01992663	0.980270596	0.962282981	0.998594447	0.034960381
PSMD11	0.043819165	1.044793403	1.022685278	1.067379455	5.93E − 05
IGF1	0.159618062	1.173062748	1.003363707	1.371463011	0.045275692
CALR	0.002182421	1.002184805	1.001012743	1.003358238	0.000256823
TAP2	− 0.03575826	0.964873516	0.931495888	0.999447141	0.046508212
KCNH2	0.042402538	1.043314368	1.023611301	1.06339669	1.31E − 05
EDNRA	0.126907424	1.135311911	1.04384756	1.23479058	0.003063118
AGTR1	0.129747552	1.138540924	0.981430523	1.32080204	0.086793836
CMTM8	0.030314707	1.030778876	0.995506182	1.067301349	0.087927298
RAC3	0.020900107	1.021120043	1.002852063	1.039720794	0.023257434
ANGPT1	0.094626463	1.099248169	0.989754074	1.22085533	0.077130021
PLXNB1	− 0.01956143	0.98062865	0.958697736	1.003061249	0.090057108
TRIM27	− 0.09275545	0.911416358	0.852085566	0.974878357	0.006917683
RBP7	0.036240038	1.036904713	1.00604832	1.068707499	0.018713692
AHNAK	0.017014449	1.01716002	1.009977752	1.024393363	2.53E − 06
IL17RE	0.05296141	1.054388955	1.004787773	1.106438691	0.031220918

$$\begin{aligned} Risk \, socre \, = & \, - \, 0.38509671 \, \times \, FCN2 \, - \, 0.00172587 \, \\ & \times \, ISG15 \, - \, 0.01992663 \, \times \, ANXA6 \, + \, 0.043819165 \\ & \times \, PSMD11 \, + \, 0.159618062 \, \times \, IGF1 \, + \, 0.002182421 \\ & \times \, CALR \, - \, 0.03575826 \, \times \, TAP2 \, + \, 0.042402538 \\ & \times \, KCNH2 \, + \, 0.126907424 \, \times \, EDNRA \, + \, 0.129747552 \\ & \times \, AGTR1 \, + \, 0.030314707 \, \times \, CMTM8 \\ & + \, 0.020900107 \, \times \, RAC3 \, + \, 0.094626463 \, \times \, ANGPT1 \\ & - \, 0.01956143 \, \times \, PLXNB1 \, - \, 0.09275545 \, \times \, TRIM27 \\ & + \, 0.036240038 \, \times \, RBP7 \, + \, 0.017014449 \, \times \, AHNAK \\ & - \, 0.05296141 \, \times \, IL17RE \\ \end{aligned}$$

### Internal and external validation of the IRGs-based prognostic index

**Fig. 6:**
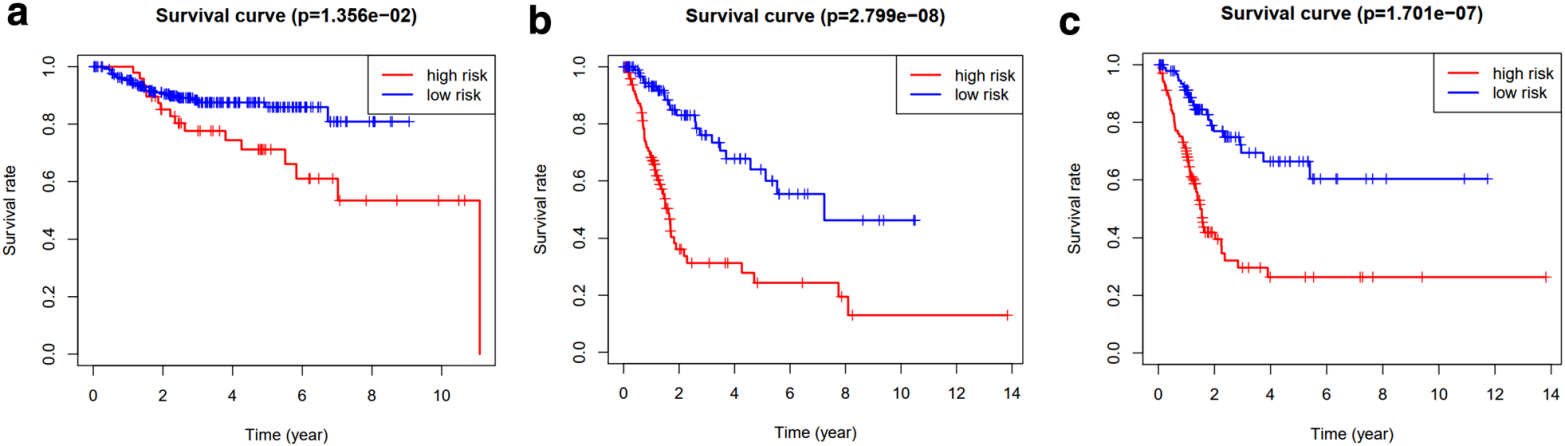
External survival validation using GEO cohort (**a**). Internal survival validation in TCGA cohort (**b**, **c**)

GEO database was used for an external validation. The difference in OS between high-risk group and low-risk group in GEO cohort was also statistically significant (P < 0.05, Fig. 6a). Internal validation was performed in train group and test group, respectively. The difference in OS between high-risk group and low-risk group was statistically significant both in train group (P < 0.05, Fig. 6b) and test group (P < 0.05, Fig. 6c).

### Prognostic evaluation and independent prognostic analysis

**Fig. 7:**
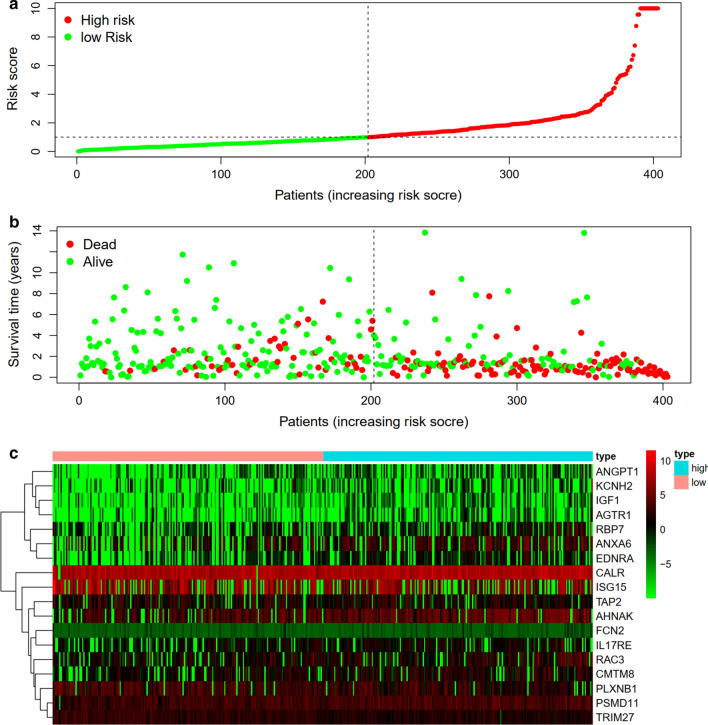
Prognostic evaluation of molecular prognostic index. The distribution of risk score (**a**). The distribution of survival time (**b**). The expression Heatmap of this index (**c**)

**Fig. 8:**
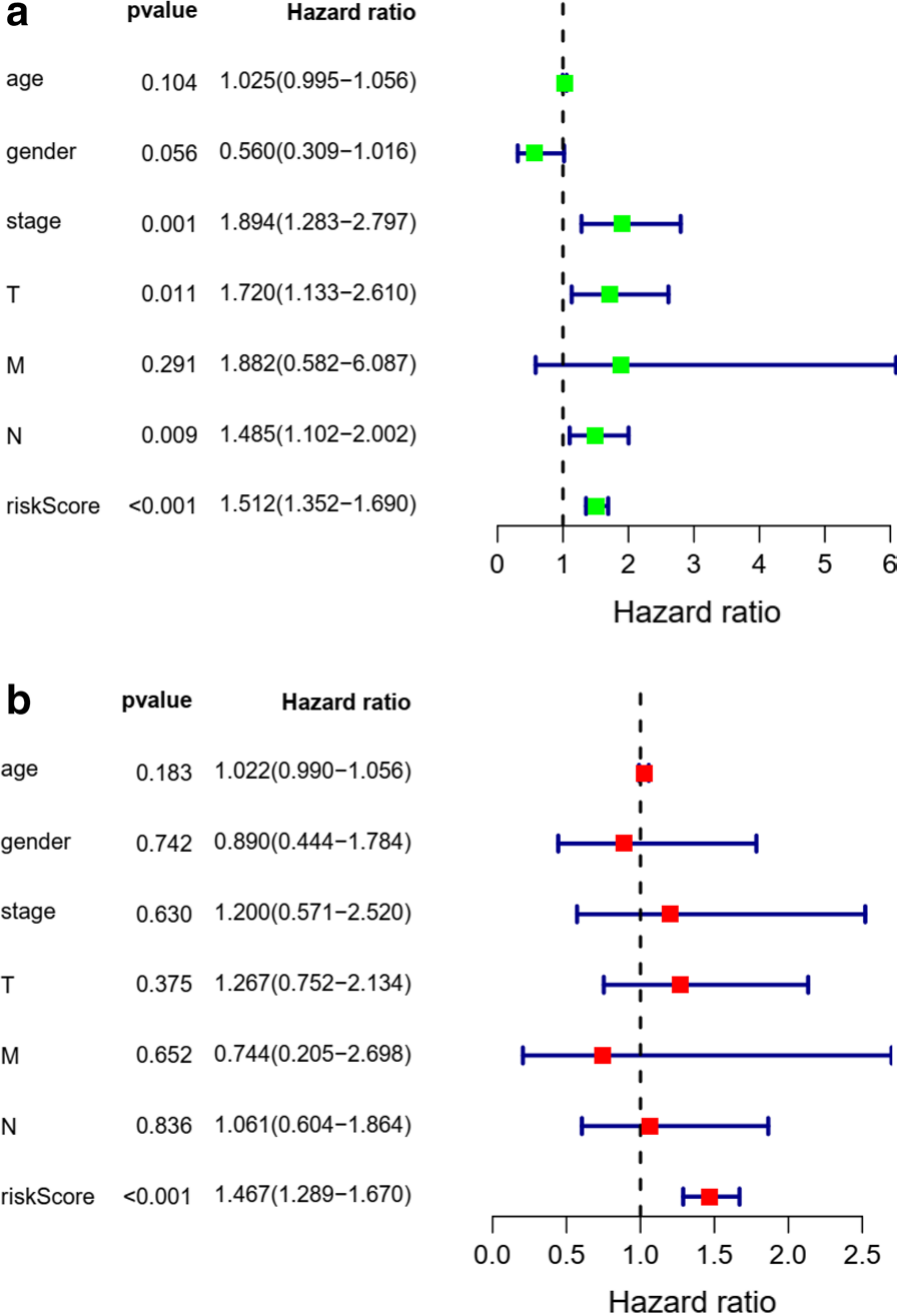
Univariate (**a**) and multivariate (**b**) independent prognostic analysis of independent risk factors for overall survival (OS) in patients with bladder cancer

**Fig. 9:**
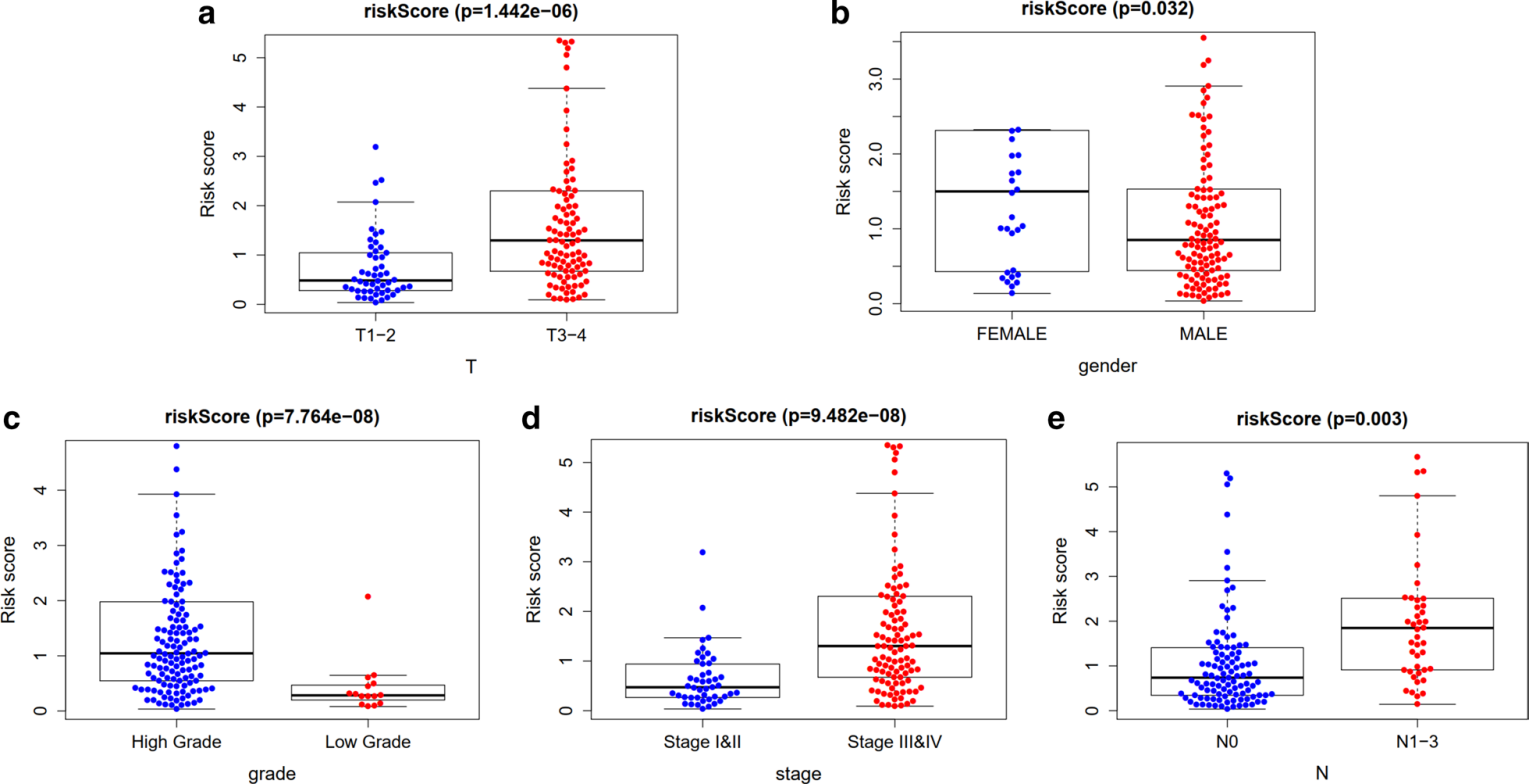
Relationship between this prognostic index and clinical characteristics. T stage (**a**); Gender (**b**); grade (**c**); stage (**d**); N stage (**e**)

The distribution of risk score and survival time were demonstrated in Fig. 7a, b. The expression heatmap of this index was demonstrated in Fig. 7c. Patients with higher risk score were associated significantly with poor prognosis. Univariate and multivariate independent prognostic analysis showed that the risk score was the only independent predictor for bladder cancer, indicating the great performance of this index (P < 0.05, Fig. 8 and Table 3). Female, high grade, stage III and IV, N1-3 and T3-4 were associated significantly with higher risk score compared with male, low grade, stage I and II, N0 and T1-2, respectively (P < 0.05, Fig. 9 and Table 4).

**Table 3:** Univariate and multivariate independent prognostic analysis of independent prognostic factor of overall survival

Variate	Univariate analysis			Multivariate analysis		
	HR	95% CI	P value	HR	95% CI	P value
age	1.025	0.995–1.058	0.104	1.022	0.990–1.056	0.183
gender	0.560	0.309–1.016	0.056	0.890	0.444–1.784	0.742
stage	1.894	1.283–2.797	0.001	1.200	0.571–2.520	0.630
T	1.720	1.130–2.620	0.011	1.267	0.752–2.134	0.375
M	1.882	0.582–6.087	0.291	0.744	0.205–2.698	0.652
N	1.485	1.102–2.002	0.009	1.061	0.604–1.864	0.836
riskScore	1.512	1.352–1.690	0.000	1.467	1.289–1.679	0.000

**Table 4:** Relationship between this prognostic index and clinical characteristics

Id	Age	Gender	Grade	Stage	T	M	N
FCN2	− 0.904 (0.368)	1.049 (0.302)	1.059 (0.291)	− 1.288 (0.201)	− 1.279 (0.204)	− 0.979 (0.373)	− 1.162 (0.252)
ISG15	0.893 (0.374)	− 0.605 (0.547)	4.947 (2.086e−06)	0.137 (0.892)	0.041 (0.967)	1.528 (0.161)	0.431 (0.667)
ANXA6	− 0.887 (0.377)	0.733 (0.467)	6.299 (3.278e−09)	− 3.604 (4.279e−04)	− 2.352 (0.020)	− 0.671 (0.527)	− 1.595 (0.116)
PSMD11	− 1.287 (0.201)	− 0.453 (0.652)	7.169 (7.037e−08)	− 4.347 (2.882e−05)	− 2.412 (0.018)	3.207 (0.009)	− 2.466 (0.016)
IGF1	0.167 (0.868)	0.814 (0.421)	2.729 (0.007)	− 3.378 (0.001)	− 2.948 (0.004)	− 0.898 (0.405)	− 1.024 (0.309)
CALR	− 0.256 (0.799)	0.451 (0.654)	4.962 (6.132e−05)	− 1.421 (0.159)	− 1.134 (0.259)	2.436 (0.047)	− 0.188 (0.851)
TAP2	1.139 (0.257)	− 0.224 (0.823)	7.519 (6.663e−12)	− 0.096 (0.924)	− 0.125 (0.901)	6.731 (1.292e−07)	− 0.024 (0.981)
KCNH2	0.413 (0.681)	0.445 (0.658)	1.097 (0.282)	− 1.578 (0.117)	− 0.391 (0.696)	− 1.041 (0.345)	− 1.049 (0.298)
EDNRA	− 1.864 (0.064)	0.58 (0.565)	5.173 (1.908e−06)	− 4.125 (6.153e−05)	− 4.279 (3.448e−05)	− 1.494 (0.193)	− 1.791 (0.077)
AGTR1	− 1.402 (0.164)	0.621 (0.539)	− 0.81 (0.431)	0.466 (0.643)	0.536 (0.593)	0.235 (0.820)	0.878 (0.382)
CMTM8	− 1.574 (0.118)	− 2.22 (0.029)	0.663 (0.517)	− 1.224 (0.224)	− 1.191 (0.236)	− 1.956 (0.105)	− 1.652 (0.103)
RAC3	− 0.403 (0.688)	1.049 (0.301)	2.818 (0.007)	− 0.553 (0.582)	0.045 (0.964)	− 1.121 (0.313)	− 1.672 (0.100)
ANGPT1	0.012 (0.990)	1.007 (0.322)	1.145 (0.265)	− 2.163 (0.032)	− 1.868 (0.064)	− 0.425 (0.686)	− 0.822 (0.413)
PLXNB1	1.423 (0.158)	− 1.088 (0.282)	− 3.054 (0.008)	3.497 (8.896e−04)	2.954 (0.004)	1.796 (0.112)	3.924 (1.352e−04)
TRIM27	1.813 (0.072)	− 2.536 (0.013)	− 2.201 (0.043)	2.488 (0.015)	2.611 (0.011)	3.608 (0.005)	2.272 (0.026)
RBP7	− 0.684 (0.495)	1.126 (0.269)	2.222 (0.028)	− 2.006 (0.047)	− 1.843 (0.068)	− 1.309 (0.247)	− 1.596 (0.118)
AHNAK	− 0.781 (0.436)	1.479 (0.148)	5.854 (2.277e−07)	− 3.813 (2.06e−04)	− 4.056 (8.06e−05)	0.93 (0.385)	− 2.199 (0.031)
IL17RE	− 1.217 (0.225)	− 0.165 (0.870)	3.215 (0.002)	− 1.686 (0.094)	− 1.347 (0.180)	− 0.755 (0.483)	− 1.667 (0.099)
riskScore	− 0.794 (0.429)	2.243 (0.032)	5.871 (7.764e−08)	− 5.649 (9.482e−08)	− 5.054 (1.442e−06)	− 2.051 (0.093)	− 3.08 (0.003)

### Relationship of the prognostic index with immune cell infiltration and tumor microenvironment

**Fig. 10:**
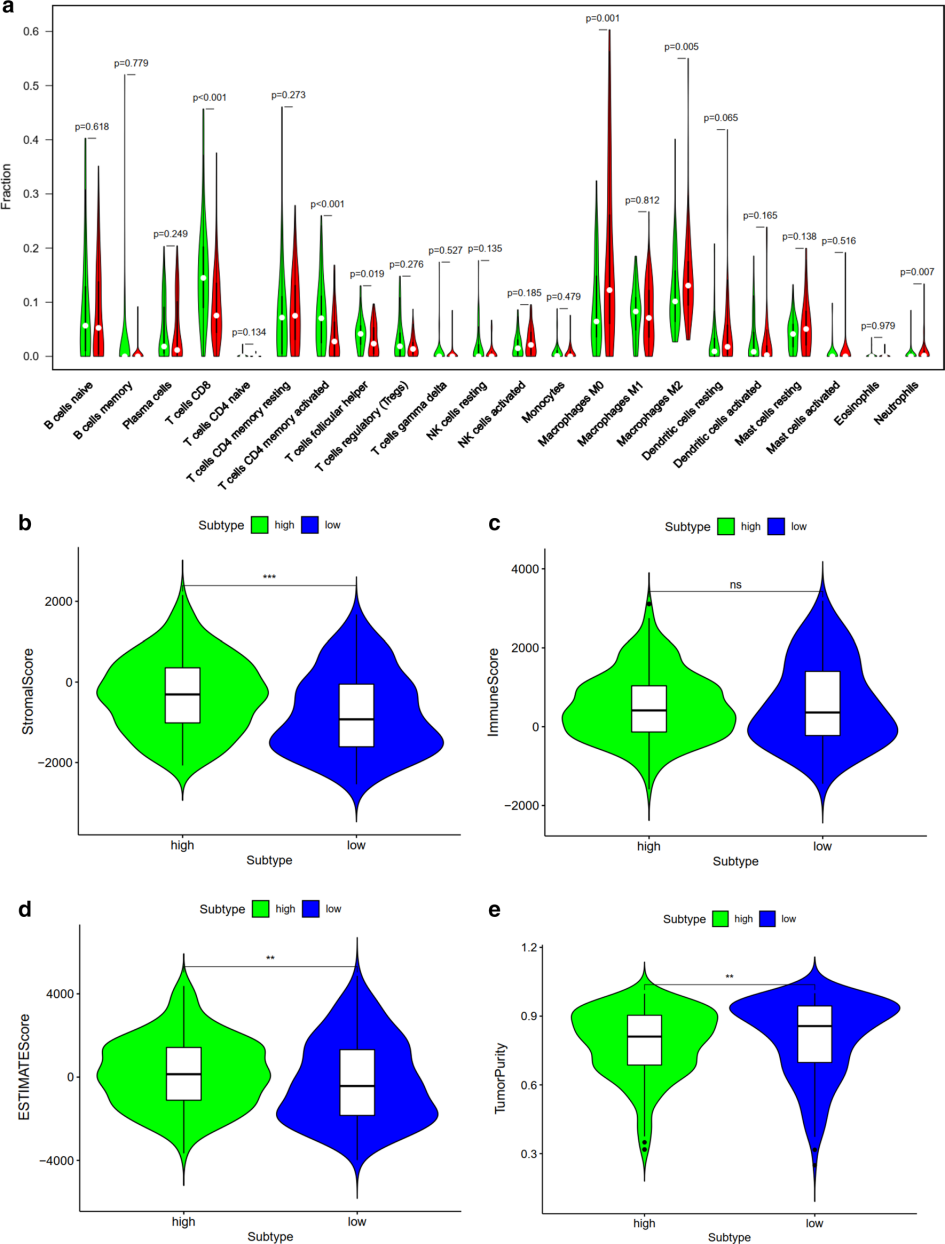
Relationship between this prognostic index and immune cell infiltration (**a**). Relationship between this prognostic index and tumor immune microenvironment. stromal score (**a**); immune score (**b**); ESTIMATE score (**c**); tumor purity (**d**)

Among the 22 subtypes of tumor-infiltrating immune cells in TIMER version 2.0 database, higher infiltrating percentage of macrophages M0, macrophages M2 and neutrophils were significantly associated with the poor prognosis of BCa, while lower infiltrating percentage of T cells CD8, T cells CD4 memory activated or T cells follicular helper were significantly associated with the poor prognosis of BCa according to the risk score derived from the molecular prognostic index (P < 0.05, Fig. 10a). Using ESTIMATE algorithm, we found that high risk score had a positive association with higher stromal score and ESTIMATE score while high risk score had a negative association with tumor purity. However, The correlation between immune score and this prognostic index was not significant (P < 0.05, Fig. 10b–e).

## Discussion

As one of the important tumors that could benefit from immunotherapy, it is of great importance to explore the specific mechanism involved in immunotherapy of BCa [11, 12]. Although there have been several prognostic models predicting the survival outcome of BCa demonstrated by previous study, few studies focus on the immune-related genes related to survival [13, 14]. This study comprehensively utilized multiple online databases to identify prognostic differentially expressed IRGs that played a vital role in survival outcome in BCa patients and explored the associated mechanism.

According to the result of univariate Cox analysis, we identified a total of 61 prognostic IRGs, among which the top 4 IRGs (including MMP9, IGF1, CXCL12 and PGF) with greatest degree score in PPI network might be of great significance for patients with BCa. MMP9, a member of the matrix metalloproteinase gene family, could be expressed to dissolve the extracellular matrix components. Fouad et al. [15] revealed that MMP-9 was significantly up-regulated in both blood and urine of BCa patients and was of great discriminatory ability in the diagnosis of BCa. Wong et al. [16] also demonstrated that MMP9 was a potential therapeutic target and prognostic biomarker that contribute to the progression of BCa. There have been several studies confirming the role of IGF-1, an anti-apoptotic peptide, in the progression of BCa. Hursting et al. [17] showed that IGF-1 pathway plays a vital role in bladder carcinogenesis in transgenic mice. Long et al. [18] reported that IGF-1/ERβ signalling plays an important role in promoting cisplatin resistance in BCa cells. However, the role of plasma IGF1 in assessing bladder cancer risk remains controversial. Zhao et al. [19] found that the plasma concentrations of IGF1 was significantly higher in BCa patients and associated with an increased risk of BCa. Nevertheless, recently, a prospective study indicated there was no association between the risk of BCa and IGF1 level in plasma [20]. This study identified IGF1 as an IRG for the first time, which might play an important role in immune response in BCa progression. The role of CXCL12 in BCa has been extensively described. It has been reported that CXCL12/CXCR4 axis plays an important role in tumor angiogenesis, and protein and mRNA levels of CXCL12 are associated with human BCa progression [21]. Batsi et al. [22] revealed that CXCL12 expression has positive association with tumor grade, irrespective of primary BCa or recurrent BCa. Yang et al. [23] demonstrated in their study that the expression of CXCR4 and its ligand CXCL12 might be in connection with depth of invasion and differentiation degree in BCa. Previous studies have showed that PGF contribute greatly to tumor growth and metastasis. Soukup et al. [24] indicated that the urine and plasma concentration of PGF were significantly increased in patients with BCa compared with those without BCa. Loredana et al. [25] summarized that PGF played a vital role in regulating tumor immune microenvironment and promoting tumor immune escape. In this study, from the perspective of tumor immunity, we identified MMP9, IGF1, CXCL12 and PGF as prognostic differentially expressed IRGs for the first time. Therefore, further study is required to explore the role of MMP9, IGF1, CXCL12 and PGF in the immune-related mechanisms and immunotherapy of BCa.

Most importantly, we developed a prognostic index based on immune-related genes, which might contribute to further understanding the specific mechanism of the effectiveness of immunotherapy and predicting clinical outcomes in patients with BCa. Previously, there have been several predicting models or indexs for BCa patients. Duan et al. [26] developed a panel for diagnosis based on three lncRNAs in serum, which was confirmed to performed better compared with urine cytology. Xion et al. [27] identified an index integrating clinical information, mRNA and miRNA for bladder urothelial carcinoma, whose AUC was distinctly increased compared with that of the RNA-alone index or clinical-alone index. Dyrskjøt et al. [28] prospectively validated a 12-gene progression score for non-muscle invasive bladder cancer (NMIBC) and found that the prognostic power of this score was superior to histopathological parameters or clinical data. Ingelmo-Torres et al. [29] constructed a predicting model based on two urinary cell microRNAs, miR-140-5p and miR-92a-3p. In this study, we developed a new prognostic index. The overall survival of patients with low risk was significantly increased compared with those with high risk. The area under curve of ROC was 0.757, suggesting the potential for this prognostic index. Further, we performed internal validation using train group and test group in TCGA cohort and external validation in GEO cohort. Both internal validation and external validation suggested the predictive power of this index. Univariate and multivariate independent prognostic analysis demonstrated that the risk score was the only independent predictor for bladder cancer, indicating the great performance of this index.

Besides, we investigated whether this index is related to immune cell infiltration, and found that higher infiltrating percentage of macrophages M0, macrophages M2 and neutrophils were significantly associated with the poor prognosis of BCa, while lower infiltrating percentage of T cells CD8, T cells CD4 memory activated or T cells follicular helper were significantly associated with the poor prognosis of BCa. It is reported that M2 macrophages play a vital role in immune responses induced by BCG against BCa [30]. Qiu et al. [31] demonstrated the regulation role of tumor-associated macrophages in BCa cell growth. Xue et al. [32] also indicated that the infiltration of M2 macrophage might be an underlying target of immunotherapy for BCa patients. Previous studies have demonstrated that tumor-infiltrating T cell landscape in bladder cancer would contribute to management decisions making, particularly immunotherapy [33]. Hou et al. [34] also revealed that the expression of PD-1 in T cell subsets provided important prognostic information in patients with BCa. Furthermore, relationship between this index and clinical characteristics were also evaluated. We found that female was associated significantly with higher risk score compared with male. It is reported that women are usually diagnosed with more advanced BCa and experience higher cancer-specific mortality [35], which is consistent with this study. One of the rational explanations is that the liver metabolizes carcinogens differently between male and female [35]. Besides, this study also revealed that patients with higher grade, higher tumor stage, higher N stage and higher T stage experienced significantly lower OS and poor prognosis. The tumor microenvironment consists of stromal cells, immune cells and tumor cells. The higher the composition of immune cells and stromal cells, the lower the proportion of tumor cells. In this study, we revealed that high risk score had a positive association with higher stromal score and ESTIMATE score but a negative association with tumor purity. However, the correlation between immune score and this prognostic index was not significant. Therefore, patients with higher risk score has higher proportion of stromal cells, and lower proportion of tumor cells. These results revealed that this index could serve as an immune status indicator for BCa patients and might contribute to understanding the mechanism of immunotherapy.

## Conclusions

Together, this study identified four prognostic hub immune-related genes, including MMP9, IGF1, CXCL12 and PGF, which might play a vital role in bladder cancer development. Besides, we developed a molecular prognostic index based on immunogenomic landscape analysis, which performed well in predicting prognosis of bladder cancer. Further researches are needed to verify the effectiveness of this index and these vital genes.

## Data Availability

All data generated or analyzed during the present study was downloaded from TCGA database, ImmPort database, GEO database and TIMER database.
